# Left Ventricular and Left Atrial Strain Characteristics in Patients with Familial Mediterranean Fever Receiving Long-Term Colchicine Therapy

**DOI:** 10.3390/diagnostics16020296

**Published:** 2026-01-16

**Authors:** Hüseyin Durak, Mustafa Çetin, Nadir Emlek, Ali Gökhan Özyıldız, Hakan Duman, Elif Ergül, Ahmet Özsipahi, Barış Dindar, Osman Cüre

**Affiliations:** 1 Department of Cardiology, Faculty of Medicine, Recep Tayyip Erdoğan University, 53100 Rize, Türkiye; 2 Department of Cardiology, Faculty of Medicine, Uşak University, 64000 Uşak, Türkiye; 3 Department of Rheumatology, Faculty of Medicine, Recep Tayyip Erdoğan University, 53100 Rize, Türkiye

**Keywords:** familial mediterranean fever, colchicine, speckle tracking, left atrial strain, left ventricular strain

## Abstract

**Background:** Familial Mediterranean fever (FMF) is a chronic autoinflammatory disorder characterized by sustained systemic inflammation that may affect cardiac structure and function. Colchicine is the cornerstone of FMF therapy and has cardiovascular benefits in inflammatory settings. **Methods:** This cross-sectional study enrolled 106 participants: 53 patients with FMF receiving long-term colchicine therapy and 53 age- and sex-matched controls. Participants underwent transthoracic echocardiography with speckle-tracking imaging. Conventional parameters and strain-derived indices of the left ventricular (LV) and left atrial (LA) function were assessed. Correlation analyses and multivariable linear regression models were used to evaluate the association between FMF presence and cardiac strain parameters. **Results:** The LV ejection fractions were comparable between the groups. The FMF group showed thinner ventricular walls and larger chamber dimensions than the control group. Patients with FMF exhibited higher LA reservoir strain, while conduit and contractile atrial contributions were reduced, as shown by lower passive and active emptying fractions and reduced LA ejection fraction. LA volumes and stiffness indices were lower in the FMF group, indicating smaller and more compliant atrial structures. Left ventricular global longitudinal strain (LVGLS) was more negative in patients with FMF, indicating preserved LV longitudinal systolic function. FMF was independently associated with LVGLS and LA strain parameters after adjusting for cardiovascular risk factors. **Conclusions:** In patients with FMF receiving long-term colchicine therapy, cardiac strain imaging showed preserved LV longitudinal function and distinct LA mechanics with preserved reservoir strain but reduced conduit and contractile function. Strain echocardiography may provide insights into cardiac involvement in well-controlled FMF, although prospective studies are needed to clarify the clinical significance of these findings.

## 1. Introduction

Familial Mediterranean fever (FMF) is the most common monogenic autoinflammatory disorder, predominantly affecting individuals of Mediterranean origin. It is characterized by recurrent episodes of fever, serositis, and arthritis resulting from mutations in the MEFV gene encoding the pyrin protein, leading to exaggerated innate immune activation and persistent subclinical inflammation, even during attack-free periods [1,2]. While the clinical manifestations of FMF are well recognized, its long-term cardiovascular implications remain unclear.

Chronic systemic inflammation is increasingly acknowledged as a key contributor to cardiovascular disease, promoting endothelial dysfunction, accelerated atherosclerosis, and adverse myocardial remodeling [3]. In patients with FMF, inflammatory markers such as C-reactive protein, interleukin-1β, and tumor necrosis factor-α may remain elevated despite clinical remission [4]. Prior studies have reported increased carotid intima–media thickness and impaired endothelial function in FMF, suggesting early vascular involvement driven by chronic low-grade inflammation and recurrent inflammatory attacks, which promote endothelial dysfunction and accelerate atherosclerotic changes [5]. However, data regarding myocardial function in FMF are inconsistent, with reports ranging from preserved cardiac performance to subclinical systolic and diastolic dysfunction.

Colchicine, a microtubule polymerization inhibitor derived from Colchicum autumnale, has been the cornerstone of FMF management for decades [6]. In addition to its efficacy in preventing inflammatory attacks and secondary amyloidosis, colchicine has attracted attention for its potential cardiovascular benefits. Large, randomized trials, including COLCOT and LoDoCo-2, have demonstrated reduced cardiovascular event rates in patients with coronary artery disease receiving low-dose colchicine [7,8]. Proposed mechanisms include the inhibition of inflammasome activation, suppression of neutrophil-mediated inflammation, modulation of endothelial function, and attenuation of fibrotic remodeling [9].

Despite these observations, the impact of long-term colchicine therapy on myocardial structure and function in patients with FMF has not been clearly defined. Speckle-tracking echocardiography (STE) enables a sensitive assessment of myocardial mechanics, including left ventricular global longitudinal strain (LVGLS) and left atrial (LA) strain components, which provide earlier detection of subclinical dysfunction than conventional echocardiographic parameters [10]. LVGLS reflects longitudinal myocardial deformation during systole, whereas LA strain assesses the reservoir, conduit, and contractile phases, providing insights into the atrial–ventricular coupling and diastolic burden [11]. These measures have demonstrated incremental diagnostic and prognostic values across various cardiovascular conditions [12].

Accordingly, the present study aimed to compare the cardiac structure and function in patients with FMF receiving stable long-term colchicine therapy and age- and sex-matched healthy controls using advanced echocardiographic techniques. We sought to characterize ventricular and atrial strain patterns in this population and explore whether long-term colchicine exposure is associated with preserved myocardial deformation parameters in the context of chronic auto-inflammation in patients with FMF.

## 2. Methods

This cross-sectional study was conducted at the rheumatology outpatient clinic of Recep Tayyip Erdoğan University Education and Research Hospital. The study involved 106 participants, including 53 patients diagnosed with FMF and 53 healthy controls matched for age and sex.

A consecutive sampling technique was used in this study to select participants. Patients with and without FMF were consecutively enrolled in the study based on the predefined inclusion criteria. Sample size was determined using G-Power version (3.1.9.7). The parameters were set as follows: alpha level (α) of 0.05, power of 0.80, and effect size of 0.5, ensuring sufficient power to detect meaningful differences. Initially, patients with FMF were recruited until the required sample size, determined by G-Power analysis, was reached. Subsequently, age- and sex-matched non-FMF patients were sequentially included.

All patients with FMF met the Tel Hashomer diagnostic criteria and had been receiving a stable colchicine regimen for at least 12 months, with doses adjusted according to clinical guidelines prior to enrollment. The Tel Hashomer criteria define FMF by the presence of at least one major criterion or two minor criteria. The major criteria include recurrent febrile attacks accompanied by serositis (peritonitis, pleuritis, or synovitis), AA amyloidosis in the absence of a predisposing condition, and a favorable response to continuous colchicine therapy. The minor criteria include recurrent febrile episodes, erysipelas-like erythema, and a family history of FMF.

The control group consisted of individuals who were age- and sex-matched to the FMF group and had no known history of colchicine use or cardiovascular or chronic inflammatory conditions.

Individuals were excluded from the study if they had a history of coronary artery disease; heart failure classified as NYHA class II–IV; moderate-to-severe valvular heart disease; atrial fibrillation or other arrhythmias; chronic kidney disease (eGFR < 60 mL/min/1.73 m^2^); pregnancy; myocarditis; cardiomyopathy; secondary hypertension; stroke; endocrine disorders; electrolyte imbalances; anemia; pulmonary thromboembolism; malignancy; active infection; or if their echocardiographic images were of insufficient quality (defined as those with insufficient resolution or artifacts affecting key measurements).

A comprehensive medical history, including information on cardiovascular risk factors and medication use, was obtained from all participants. Following physical examination, venous blood samples were obtained by venipuncture after an overnight 12 h fast for laboratory analyses, including complete blood count, fasting glucose, lipid profile, renal function tests, total protein, albumin, and C-reactive protein.

Transthoracic echocardiography was performed in all participants using a Vivid E95 system (Ultra Edition; GE Healthcare, Horten, Norway) equipped with an M5Sc transducer. All studies were conducted by a single experienced cardiologist with a minimum of five years of expertise in echocardiographic imaging, who was blinded to the group allocation, in accordance with the current guidelines of the American Society of Echocardiography (ASE).

Conventional measurements included left ventricular (LV) dimensions, interventricular septum (IVS), posterior wall (PW) thickness, and LV ejection fraction (LVEF) which was calculated using the modified Simpson’s method. Mitral inflow velocities (E and A waves), E/A ratio, and tissue Doppler parameters (E’, A’, and S’) were also recorded. Right ventricular function was assessed by measuring the tricuspid annular plane systolic excursion (TAPSE), right ventricular fractional area change (RVFAC), and tricuspid S’ wave velocity. Systolic pulmonary artery pressure (SPAP) was estimated from tricuspid regurgitation velocity using the modified Bernoulli equation.

STE analysis was performed offline using vendor-specific software (EchoPAC version 204, revision 73; GE Healthcare). Echocardiographic acquisitions were obtained using a GE ultrasound platform (Application software version 204; platform VS60N/70N and VE80/90/95 series).

LVGLS was calculated from apical four-, two-, and three-chamber views by averaging peak systolic longitudinal strain values from an 18-segment model, with strain analysis referenced to the onset of the QRS complex.

LA strain analysis was performed using apical four- and two-chamber views, with manual tracing of the LA endocardial border while excluding the pulmonary veins and the LA appendage. LA reservoir, conduit, and contractile strain components were derived using the QRS onset as the zero-reference point.

Frame rates for strain analysis ranged between 60 and 90 frames per second. Image quality criteria for STE included adequate endocardial border definition throughout the cardiac cycle and the absence of significant foreshortening. Images not meeting these criteria were excluded from strain analysis. Acquisition settings and image quality criteria were applied consistently across both study groups to minimize technical variability in strain-derived parameters.

LA volumes were measured at the maximum (Vmax), minimum (Vmin), and pre-atrial contraction phases (Vpre-A) (at the onset of the P-wave on ECG). These measurements were used to calculate the LA ejection fraction (LAEF), expansion index, passive and active emptying fractions, and stiffness index using the following formulas:

LAEF = [(Vmax − Vmin)/Vmax] × 100;

LA passive emptying fraction = [Vmax-Vpre-A/Vmax] × 100;

LA active emptying fraction = [Vpre-A − Vmin/Vpre-A] × 100;

LA expansion index = [(Vmax − Vmin)/Vmin] × 100;

LA stiffness index = E/E’ ratio/LA reservoir strain.

### 2.1. Ethical Approval and Informed Consent

The study protocol was approved by the Ethics Committee of Recep Tayyip Erdoğan University (Recep Tayyip Erdoğan University Non-Interventional Clinical Research Ethics Committee Chair, Date: 12 March 2025, Document Number: E-40465587-050.01.04-1395). All procedures were conducted in accordance with the principles outlined in the Declaration of Helsinki. Written informed consent was obtained from all participants prior to their inclusion in the study.

### 2.2. Statistical Analysis

All statistical analyses were performed using SPSS version 19.0 (SPSS Inc., Chicago, IL, USA). Continuous variables are expressed as mean ± standard deviation or median and interquartile range, depending on the data distribution, as assessed using the Kolmogorov–Smirnov test. Categorical variables are expressed as counts and percentages. Between-group comparisons were conducted using the independent samples *t*-test or Mann–Whitney U test for continuous variables and the Chi-square or Fisher’s exact test for categorical variables. Correlation analyses were performed using the Pearson correlation coefficient.

Multivariable linear regression analyses were conducted using the enter method, whereby all covariates deemed clinically relevant or significant in the univariable analyses were entered simultaneously into the model to assess independent associations.

The inter- and intra-observer reproducibility of STE measurements was assessed in a randomly selected subset of 20 participants. LVGLS and LA strain parameters, including reservoir, conduit, and contractile strains, were reanalyzed offline. For intra-observer variability, the measurements were repeated by the same observer at least two weeks apart, blinded to the initial results. For inter-observer variability, a second experienced observer independently analyzed the same dataset. Reproducibility was evaluated using intraclass correlation coefficients (ICC) based on a two-way random-effects model with absolute agreements.

Statistical significance was set at *p* < 0.05.

## 3. Results

### 3.1. Baseline Clinical and Laboratory Characteristics

This comparative cross-sectional study included 106 participants: 53 patients with FMF receiving long-term colchicine therapy and 53 age- and sex-matched healthy controls. The baseline demographic, clinical, and laboratory characteristics of the study groups are summarized in Table 1.

The mean duration of FMF was 15.13 ± 11.2 years. Patients were receiving colchicine at a mean daily dose of 1.39 ± 0.29 mg.

The FMF patient cohort exhibited a lower prevalence of traditional cardiovascular risk factors than the control group. Specifically, smoking (20.8% vs. 40.4%, *p* = 0.024), diabetes mellitus (DM) (3.8% vs. 18.9%, *p* = 0.014), and hypertension (HT) (7.5% vs. 22.6%, *p* = 0.028) were significantly less frequent. Consistent with the lower burden of cardiovascular risk factors, patients with FMF were less frequently prescribed cardiovascular medications, including angiotensin-converting enzyme inhibitors, angiotensin receptor blockers, beta-blockers, diuretics, statins, and antidiabetic agents (all *p* < 0.05).

The laboratory parameters were consistent with the differences in metabolic characteristics between the groups. Fasting glucose levels were significantly lower in the FMF group (90.2 ± SD mg/dL vs. 105.2 ± SD mg/dL, *p* = 0.004), as were total cholesterol concentrations (193.1 ± SD mg/dL vs. 211 ± SD mg/dL, *p* = 0.041). In addition, lymphocyte counts were significantly lower in patients with FMF than in those without (2.07 ± SD × 10^3^/μL vs. 2.4 ± SD × 10^3^/μL, *p* = 0.012).

### 3.2. Conventional Echocardiographic Findings

**Figure 1:**
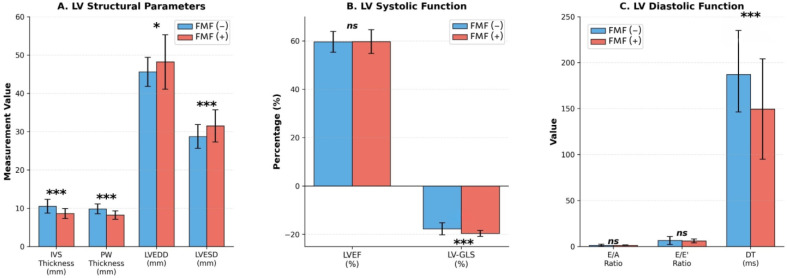
Comparative analysis of left ventricular structural, systolic, and diastolic parameters between FMF patients treated with colchicine and healthy controls. IVS = interventricular septum; PW = posterior wall; LVEDD = left ventricular end-diastolic dimension; LVESD = left ventricular end-systolic dimension; LVEF = left ventricular ejection fraction; LV-GLS = left ventricular global longitudinal strain; E/A = ratio of early to late diastolic mitral inflow velocity; E/E’ = ratio of early mitral inflow velocity to early diastolic mitral annular velocity; DT = deceleration time. * *p* < 0.05, *** *p* < 0.001, ns = not significant.

Standard two-dimensional and Doppler echocardiographic assessments revealed several differences between the groups. Although no clinically overt cardiac disease was present, patients with FMF demonstrated distinct structural and functional echocardiographic characteristics. The conventional echocardiographic differences between patients with FMF and controls are summarized in Figure 1 and detailed in Table 2. 

#### 3.2.1. Left Ventricular Structure

Patients with FMF had significantly thinner LV walls than controls, as reflected by reduced IVS thickness (8.6 ± 1.3 mm vs. 10.5 ± 1.8 mm, *p* < 0.001) and PW thickness (8.2 ± 1.1 mm vs. 9.8 ± 1.3 mm, *p* < 0.001). In contrast, LV chamber dimensions were larger in the FMF group, with increased LV end-diastolic diameter (LVEDD: 48.2 ± 7.1 mm vs. 45.6 ± 3.8 mm, *p* = 0.024) and end-systolic diameter (LVESD: 31.5 ± 4.2 mm vs. 28.7 ± 3.1 mm, *p* < 0.001). Despite these structural differences, the LVEF remained preserved and comparable between the groups.

#### 3.2.2. Diastolic Function

Assessment of LV diastolic function demonstrated differences in the filling parameters between the groups. The mitral inflow deceleration time was significantly shorter in the FMF group than in the control group (149.4 ± 54.6 ms vs. 187.2 ± 40.8 ms, *p* < 0.001). In addition, estimated SPAP was lower in patients with FMF (15.06 ± 8.1 mmHg vs. 19.2 ± 4.2 mmHg, *p* = 0.016). 

#### 3.2.3. Speckle-Tracking Echocardiography

##### Left Ventricular Strain

STE revealed differences in LV systolic deformation between the groups. LVGLS was significantly more negative in the FMF group than in the control group (−19.6 ± 1.2% vs. −17.7 ± 2.5%, *p* < 0.001).

### 3.3. Left Atrial Strain and Functional Analysis

**Figure 2:**
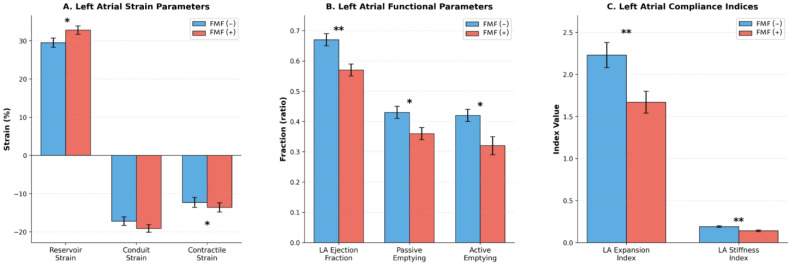
Comparative analysis of left atrial strain, functional parameters, and compliance indices between FMF patients treated with colchicine and healthy controls. LA = left atrial; * *p* < 0.05, ** *p* < 0.01.

The associations between FMF presence and cardiac strain parameters are illustrated in Figure 2 and summarized in Table 3.

Analysis of LA phasic function revealed significant differences between FMF patients and controls. LA reservoir strain was significantly higher in the FMF group compared with controls (34.2 ± 12.7% vs. 29.1 ± 9.7%, *p* = 0.039). Contractile strain magnitude was also significantly greater in FMF patients (–14.7 ± 6.6% vs. –11.6 ± 4.7%, *p* = 0.021), whereas conduit strain did not differ significantly between the groups (–19.4 ± 6.1% vs. –17.6 ± 7.8%, *p* = 0.215).

In contrast, volumetric LA functional parameters were lower in the FMF group. LAEF was significantly reduced in FMF patients compared with controls (0.57 ± 0.13 vs. 0.66 ± 0.09, *p* < 0.001). Similarly, the LA expansion index was lower in the FMF group (1.67 ± 1.01 vs. 2.22 ± 0.96, *p* = 0.005). Passive emptying fraction (0.36 ± 0.13 vs. 0.42 ± 0.14, *p* = 0.043) and active emptying fraction (0.32 ± 0.17 vs. 0.41 ± 0.13, *p* = 0.010) were also significantly lower in FMF patients. Additionally, the LA stiffness index was significantly lower in the FMF group than in controls (0.18 ± 0.09 vs. 0.21 ± 0.12, *p* = 0.005).

### 3.4. Correlation Analysis

**Figure 3:**
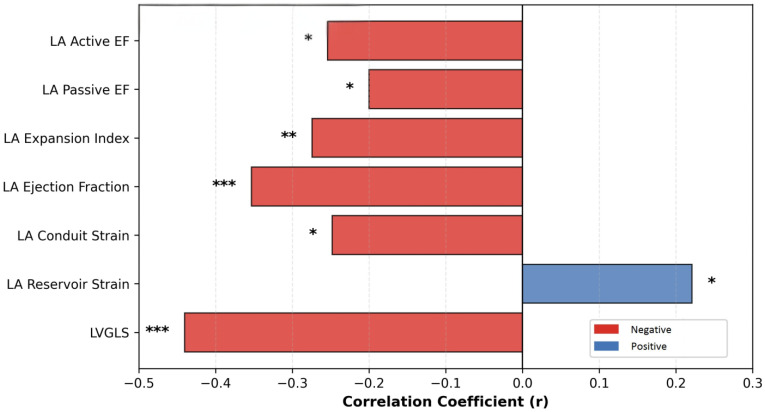
Correlation analysis demonstrating the associations between FMF presence and left ventricular and left atrial strain-derived parameters. Bars represent correlation coefficients (r). Negative correlations are shown in red and positive correlations in blue. FMF presence was associated with more negative LVGLS values and altered left atrial mechanics, including reservoir, conduit, and contractile function indices. * *p* < 0.05, ** *p* < 0.01, *** *p* < 0.001.

Univariate correlation analysis identified several significant associations between clinical variables and cardiac strain indices (Figure 3).

Correlation analyses were performed to examine the relationships between clinical variables and echocardiographic functional parameters, as summarized in Table 4 and Table 5. FMF status was significantly correlated with multiple cardiac indices. Specifically, FMF was correlated with more negative LVGLS values (r = −0.440, *p* < 0.001). In addition, FMF status was positively correlated with LA reservoir strain (r = 0.221, *p* = 0.039) and negatively correlated with conduit strain (r = −0.248, *p* = 0.021). FMF was also inversely correlated with the LAEF (r = −0.353, *p* < 0.001).

Further analyses demonstrated significant negative correlations between FMF status and volumetric indices of LA function, including the LA expansion index (r = −0.274, *p* = 0.005), passive emptying fraction (r = −0.200, *p* = 0.043), and active emptying fraction (r = −0.254, *p* = 0.010).

Traditional cardiovascular risk factors and metabolic variables were correlated with cardiac functional parameters. DM, fasting glucose levels, and HT were significantly correlated with indices of LA stiffness and active emptying function. The use of cardiovascular medications demonstrated variable correlations with the LA mechanical indices.

#### 3.4.1. Multivariable Regression Analyses

**Figure 4:**
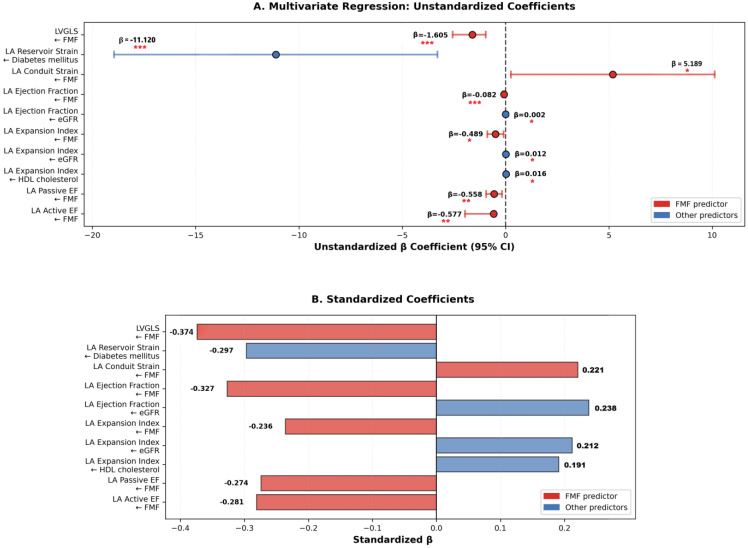
Multivariate linear regression analyses identifying independent predictors of cardiac strain parameters. (**A**) Unstandardized regression coefficients (β) with 95% confidence intervals for FMF status and other covariates. (**B**) Standardized β coefficients demonstrating the relative contribution of each predictor to the respective strain parameters. FMF status remained independently associated with LVGLS and multiple left atrial strain-derived indices after adjustment for cardiovascular risk factors, metabolic parameters, and medication use. Arrows indicate the direction of the regression relationship, pointing from the predictor to the outcome variable. * *p* < 0.05, ** *p* < 0.01, *** *p* < 0.001.

Multivariate linear regression analyses were performed to identify independent associations between the FMF status and cardiac strain parameters, while accounting for potential confounders. The independent predictors identified by these models are summarized in Figure 4 and Table 6, respectively.

#### 3.4.2. Independent Predictors of LV Strain

FMF was an independent variable associated with LVGLS (β = −1.605, *p* < 0.001) after adjustment for cardiovascular risk factors, metabolic parameters, and medication use.

#### 3.4.3. Independent Associations with LA Strain

FMF was independently associated with multiple LA strain-derived parameters, including LA conduit strain (β = 5.189, *p* = 0.039), LA ejection fraction (β = −0.082, *p* < 0.001), LA expansion index (β = −0.489, *p* = 0.014), LA passive ejection fraction (β = −0.558, *p* = 0.005), and LA active ejection fraction (β = −0.577, *p* = 0.004).

#### 3.4.4. Additional Independent Associations

DM was independently associated with LA reservoir strain. The estimated glomerular filtration rate (eGFR) was independently associated with LAEF and expansion index.

These multivariate analyses indicated that the association between FMF and cardiac strain parameters persisted after adjustment for traditional cardiovascular risk factors and metabolic variables.

#### 3.4.5. Reproducibility of Strain Measurements

Inter- and intra-observer reproducibility analyses were performed on a randomly selected subset of 20 participants in this study. The intra-observer ICCs ranged from 0.86 to 0.93, and the inter-observer ICCs ranged from 0.84 to 0.91 for LVGLS, as well as for the left atrial reservoir, conduit, and contractile strain parameters, indicating excellent reproducibility of the measurements.

## 4. Discussion

This study provides detailed strain echocardiographic data on the cardiac structure and function of patients with FMF receiving long-term colchicine therapy. Our findings demonstrated distinct patterns of LV systolic deformation, ventricular geometry, and LA strain parameters. These observations differ from the commonly reported subclinical myocardial impairment in chronic inflammatory conditions and should be interpreted cautiously, considering the cross-sectional design and clinical characteristics of the study population.

The present study identified three principal findings. First, patients with FMF receiving long-term colchicine therapy exhibited more negative LVGLS values than age- and sex-matched controls. Second, patients with FMF showed distinct differences in LV geometry, characterized by thinner myocardial walls and larger chamber dimensions. Third, LA mechanics differed between the groups, with a relatively higher reservoir strain accompanied by lower indices of atrial contractile function.

The most prominent finding of this study was the more negative LVGLS observed in patients with FMF than in controls, which remained independently associated with FMF in the multivariable analysis. LVGLS is a superior indicator of subclinical systolic dysfunction compared to conventional ejection fraction, particularly in inflammatory conditions affecting subendocardial fibers [13]. This observation shows both agreement and differences with prior studies on cardiac involvement in FMF and other inflammatory conditions. Previous echocardiographic studies on FMF have reported varied results regarding systolic function. Pamukçu et al. documented a reduction in right ventricular global longitudinal strain in patients with FMF compared to controls [14], while Çalışkan et al. identified impaired diastolic function in patients with FMF [15]. Using strain-based assessment, this study showed preserved LV systolic strain parameters in patients with FMF on long-term colchicine therapy with controlled disease activity.

Colchicine, the cornerstone of FMF therapy, exerts anti-inflammatory effects via multiple pathways. Evidence from trials such as COLCOT and LoDoCo2 has shown that low-dose colchicine reduces major cardiovascular events in patients with coronary artery disease [7,8]. These mechanisms include NOD-like receptor family pyrin domain containing 3 (NLRP3) inflammasome inhibition, suppression of pro-inflammatory cytokines, reduction of oxidative stress, disruption of microtubule polymerization, and improvement in coronary microvascular function [16,17]. In an in vitro fibroblast model, Astiawati et al. showed that hypoxia induced activation of the NLRP3 inflammasome and that colchicine treatment significantly reduced interleukin-1β expression while attenuating apoptosis-associated speck-like protein containing a CARD (ASC)–NLRP3 colocalization, suggesting a potential inhibitory effect of colchicine on inflammasome-mediated inflammatory signaling in cardiac fibroblasts [18]. Handari et al. found that colchicine was associated with increased interleukin-10 expression under hypoxic conditions in fibroblasts and in patients with acute myocardial infarction, indicating a possible enhancement of the anti-inflammatory response and attenuation of post-infarction inflammation [19]. In a multicenter, randomized, placebo-controlled trial, Astiawati et al. demonstrated that colchicine treatment was associated with significant reductions in N-terminal pro–B-type natriuretic peptide, caspase-1, transforming growth factor-β, and galectin-3 levels in patients with acute myocardial infarction, reflecting attenuation of post-infarction inflammatory and fibrotic activity, although no significant improvement in short-term echocardiographic parameters was observed [20]. Handari et al. examined fibrosis-related biomarkers in patients with ST-segment elevation acute coronary syndrome treated with colchicine and found that colchicine was associated with reduced levels of procollagen III N-terminal propeptide in patients undergoing delayed percutaneous coronary intervention, indicating a possible role in modulating post-infarction fibrotic remodeling [21]. In patients with FMF receiving long-term colchicine therapy, sustained attenuation of inflammatory signaling may preserve myocardial deformation parameters. This may explain why the LV strain indices in this cohort differed from those in other inflammatory conditions while remaining consistent with the study design. Similar benefits have been observed in other inflammatory disorders, such as rheumatoid arthritis and systemic lupus erythematosus, where anti-inflammatory treatment has been associated with improvements in myocardial strain and reductions in cardiovascular risk [22,23]. Chronic inflammatory activity in FMF may trigger adaptive cardiac responses similar to preconditioning phenomena. When suppressed by colchicine therapy, recurrent inflammatory stimuli may activate protective mechanisms, including heat shock proteins and antioxidant defenses. These adaptive responses may enhance myocardial resilience and explain the preserved myocardial deformation in this cohort.

The FMF cohort exhibited a more favorable cardiovascular risk profile than the controls, with lower prevalences of smoking, DM, and HT, as well as more favorable metabolic parameters, including fasting glucose and total cholesterol levels. This imbalance may partly reflect selection-related factors, as patients with FMF are typically under regular medical follow-up and may receive closer preventive care. Importantly, the association between FMF and LVGLS persisted after adjusting for major cardiovascular risk factors in multivariable analyses, suggesting that differences in baseline cardiometabolic risk alone do not fully account for the observed strain findings.

Patients with FMF exhibited thinner IVS and PW thicknesses in combination with larger LV chamber dimensions. Importantly, these differences are likely influenced by the lower prevalence of HT, DM, and related cardiometabolic stressors in the FMF cohort, all of which are well-established drivers of LV hypertrophic remodeling [24]. In parallel, chronic inflammation is known to promote adverse myocardial remodeling through hypertrophy, fibrosis, and diastolic dysfunction; therefore, sustained suppression of inflammatory activity with long-term colchicine therapy may limit the development of inflammation-related hypertrophic and fibrotic stimuli. In this context, the thinner ventricular walls observed in patients with FMF may reflect the relative absence of pathological remodeling. Finally, the coexistence of larger chamber dimensions with more negative LVGLS values may represent a favorable geometric–functional coupling that allows the maintenance of effective cardiac performance without overt systolic impairment, consistent with a pattern of physiological adaptation rather than maladaptive remodeling [25]. These interpretations should be considered within the limitations of the cross-sectional design and the absence of direct characterization of the myocardial tissue.

This study demonstrated heterogeneous LA functional changes, with preserved reservoir strain but reduced conduit and contractile functions. The higher LA reservoir strain in FMF patients suggests preserved atrial filling during ventricular systole [26], possibly due to favorable LV systolic deformation, increased atrial compliance, or adaptive mechanisms that maintain ventricular preload. The coexistence of higher LA reservoir strain with lower conduit and contractile functional contributions observed in patients with FMF receiving long-term colchicine therapy reflects a distinct pattern of atrial–ventricular interaction rather than advanced atrial dysfunction. Although conduit and contractile strain values tended to be more negative, the volume-based indices of atrial function, including the LAEF and passive and active emptying fractions, were reduced, indicating a diminished atrial contribution to LV filling. This apparent dissociation highlights the difference between atrial myocardial deformation and the effective volumetric transfer.

In the presence of a smaller and more compliant LA with reduced atrial stiffness, preserved or enhanced reservoir strain likely reflects maintained atrial compliance and efficient atrial filling. Conversely, the reduced reliance on the conduit and active contractile phases suggests that LV filling can be adequately achieved without substantial passive or booster atrial contributions. This pattern is consistent with a hemodynamic state in which atrial contractile reserve is not required, rather than intrinsic atrial failure or pathological remodeling. In FMF, inflammatory activity may preferentially affect the atrial tissue, contributing to reduced contractile function. Colchicine may exert different effects on the ventricular and atrial myocardium, with atrial function being more sensitive to colchicine-related modulation of the cellular contractile apparatus. The reduction in atrial contractile indices may reflect a hemodynamic adaptation to preserved ventricular systolic deformation, representing an efficient LA-LV coupling strategy that maintains effective ventricular filling while limiting the atrial workload.

Regarding LA mechanics, previous studies on various cardiovascular conditions have established that the reservoir, conduit, and contractile strain components provide complementary insights into LA-LV coupling and diastolic function [27,28,29]. In the present study, the dissociation between the preserved reservoir strain and reduced contractile indices represents a pattern that has not been frequently emphasized in previous reports. This finding may reflect disease- or treatment-related influences on atrial remodeling in FMF and highlights the value of comprehensive strain analysis in characterizing chamber-specific functional adaptation.

The altered LA mechanical patterns observed in patients with FMF warrant careful consideration in future studies. LA dysfunction has been implicated in the development of atrial fibrillation and heart failure with a preserved EF. Prospective longitudinal studies are required to determine whether these atrial mechanical alterations are associated with modified arrhythmic or heart failure risk in FMF and to clarify their clinical significance.

### Limitations

The present study had several limitations. First, comprehensive MEFV genotyping data were not available for all participants because genetic testing was not routinely performed at the time of enrollment. Given that MEFV mutations may influence disease severity, inflammatory burden, and potential cardiovascular involvement, the absence of complete genotype information limits our ability to explore genotype–phenotype associations or adjust for genetic heterogeneity. Therefore, the present findings should be interpreted independently of specific MEFV mutation subtypes, and future studies incorporating systematic genetic characterization are warranted to clarify the impact of MEFV genotypes on the myocardial and atrial mechanical properties.

Second, although the control group was matched for age and sex, it had a higher burden of traditional cardiovascular risk factors, which may have influenced some observed differences. Although multivariable adjustment was performed and FMF status remained independently associated with several strain-derived parameters, the findings should be interpreted as associative rather than causal.

Third, inflammatory activity was assessed using a single-time-point CRP measurement, which may not fully reflect the fluctuating inflammatory burden characteristics of FMF. Transient changes in inflammatory activity or subclinical inflammation may have influenced the myocardial and atrial strain parameters but could not be captured in the present analysis. The absence of additional biomarkers, such as serum amyloid A or proinflammatory cytokines, limited a more detailed evaluation of the relationship between inflammatory activity and cardiac deformation indices.

Additional limitations include the cross-sectional study design, which precludes inference regarding causality and temporal relationships; the lack of a detailed assessment of colchicine adherence; the absence of a systematic evaluation of disease activity and attack frequency; and the lack of cardiac magnetic resonance imaging, which would have provided complementary information on myocardial fibrosis, edema, and tissue characterization. Finally, the modest sample size may have limited statistical power for subgroup analyses and the detection of smaller effect sizes.

## 5. Conclusions

In patients with FMF receiving long-term colchicine therapy, strain echocardiography demonstrated preserved LV systolic deformation and distinct patterns of LA mechanical function compared with those of matched controls. These findings suggest that myocardial deformation parameters may remain preserved in well-controlled FMF. The observed alterations in LA mechanics highlight the potential chamber-specific influences of disease and treatment. Within the limitations of a cross-sectional design, strain imaging provides valuable insights into the subclinical cardiac characteristics of patients with FMF. Further prospective studies are required to clarify the clinical significance and temporal evolution of these observations.

## Figures and Tables

**Table 1:** Baseline Demographic and Clinical Characteristics of Study Participants.

Parameter	FMF (–) (*n* = 53)	FMF (+) (*n* = 53)	*p*-Value
Age (years)	40.5 ± 10.7	40.4 ± 11.1	0.915
Sex (male, *n*, %)	23 (43.4%)	23 (43.4%)	1.000
Body Mass Index (kg/m^2^)	27.6 ± 5.6	27.7 ± 4.7	0.955
**Smoking (*n*, %)**	**21 (40.4%)**	**11 (20.8%)**	**0.024**
**Diabetes Mellitus (*n*, %)**	**10 (18.9%)**	**2 (3.8%)**	**0.014**
**Hypertension (*n*, %)**	**12 (22.6%)**	**4 (7.5%)**	**0.028**
Hyperlipidemia (*n*, %)	27 (50.9%)	20 (37.7%)	0.120
**ACE inhibitor/ARB use (*n*, %)**	**10 (18.9%)**	**2 (3.8%)**	**0.014**
**Beta-blocker use (*n*, %)**	**13 (24.5%)**	**0 (0%)**	**<0.001**
Calcium Channel Blocker use (*n*, %)	5 (9.4%)	3 (5.7%)	0.358
**Diuretic use (*n*, %)**	**5 (9.4%)**	**0 (0%)**	**0.028**
**Statin use (*n*, %)**	**10 (18.9%)**	**2 (3.8%)**	**0.014**
**Oral antidiabetic use (*n*, %)**	**8 (15.1%)**	**1 (1.9%)**	**0.016**
White Blood Cell Count (×10^3^/μL)	7.7 ± 1.9	7.3 ± 2.1	0.427
Neutrophils (×10^3^/μL)	4.6 ± 1.5	4.6 ± 2.1	0.896
**Lymphocytes (×10^3^/μL)**	**2.4 ± 0.6**	**2.07 ± 0.5**	**0.012**
**Hemoglobin (g/dL)**	**13.9 ± 1.8**	**13.4 ± 1.7**	**0.012**
**Total Protein (g/dL)**	**7.2 ± 0.69**	**7.5 ± 0.43**	**0.015**
Albumin (g/dL)	4.4 ± 0.7	4.4 ± 0.3	0.964
Serum Creatinine (mg/dL)	0.76 ± 0.18	0.77 ± 0.23	0.817
Estimated GFR (mL/min/1.73 m^2^)	106 ± 16.7	102 ± 18.6	0.264
**Glucose (mg/dL)**	**105.2 ± 36.1**	**90.2 ± 9.6**	**0.004**
**Total Cholesterol (mg/dL)**	**211 ± 52.6**	**193.1 ± 38.5**	**0.041**
Triglycerides (mg/dL)	163.3 ± 127.4	134.8 ± 84.5	0.184
HDL-C (mg/dL)	49.9 ± 13.9	47.3 ± 11.2	0.406
LDL-C (mg/dL)	135.6 ± 45.6	120.1 ± 33.5	0.051
C-Reactive Protein (mg/L)	3.5 (2.4–6.5)	4 (2–8.6)	0.361

Statistically significant parameters are highlighted in bold. ACE, angiotensin converting enzyme; ARB, angiotensin receptor blocker; FMF, familial Mediterranean fever; GFR, glomerular filtration rate; HDL-C, high density lipoprotein cholesterol; LDL-C, low density lipoprotein cholesterol.

**Table 2:** Conventional Echocardiographic Parameters in FMF Patients and Controls.

Parameter	FMF (−) (*n* = 53)	FMF (+) (*n* = 53)	*p*
Left Ventricular Ejection Fraction (%)	59.6 ± 4.3	59.7 ± 4.9	0.891
**Interventricular Septum Thickness (mm)**	**10.5 ± 1.8**	**8.6 ± 1.3**	**<0.001**
**Posterior Wall Thickness (mm)**	**9.8 ± 1.3**	**8.2 ± 1.1**	**<0.001**
**LV End-Diastolic Diameter (mm)**	**45.6 ± 3.8**	**48.2 ± 7.1**	**0.024**
**LV End-Systolic Diameter (mm)**	**28.7 ± 3.1**	**31.5 ± 4.2**	**<0.001**
**LV Global Longitudinal Strain (%)**	**−17.7 ± 2.5**	**−19.6 ± 1.2**	**<0.001**
Total Atrial Conduction Time (s)	0.59 ± 0.13	0.55 ± 0.16	0.096
Mitral E-wave Velocity (cm/s)	78.1 ± 22	81.9 ± 18.8	0.350
Mitral A-wave Velocity (cm/s)	69.8 ± 16.2	65.8 ± 18.1	0.237
Lateral E’ (cm/s)	13.7 ± 4.3	14.3 ± 3.2	0.406
Lateral A’ (cm/s)	10.1 ± 2.1	9.6 ± 2.5	0.263
Lateral S’ (cm/s)	9.9 ± 2.1	9.6 ± 3.01	0.484
E/A Ratio	1.28 ± 1.14	1.28 ± 0.3	0.990
E/E’ Ratio	6.5 ± 4.2	6.01 ± 2.1	0.468
**Deceleration Time (ms)**	**187.2 ± 40.8**	**149.4 ± 54.6**	**<0.001**
Aortic Root Diameter (Systole) (mm)	30.8 ± 4.7	30.5 ± 2.9	0.657
Aortic Root Diameter (Diastole) (mm)	29.1 ± 4.7	28.9 ± 2.7	0.630
TAPSE (mm)	23.3 ± 3.4	23.0 ± 2.8	0.827
RVFAC (%)	44.5 ± 8.9	43.6 ± 6.7	0.575
Right Ventricular Systolic Velocity (S’) (cm/s)	14.4 ± 2.6	14.1 ± 2.6	0.502
**SPAP (mmHg)**	**19.2 ± 4.2**	**15.06 ± 8.1**	**0.016**

Statistically significant parameters are highlighted in bold. Normal reference values for LVGLS were considered approximately −18% to −22% in adults with preserved systolic function. Reference ranges are provided for descriptive purposes only and were not used as diagnostic thresholds. LV, left ventricular; SPAP, systolic pulmonary artery pressure; TAPSE, tricuspid annular plane systolic excursion; RVFAC, right ventricular fractional area changing.

**Table 3:** Left Atrial Structure and Function Parameters.

Parameter	FMF (−) (*n* = 53)	FMF (+) (*n* = 53)	*p*
**Left Atrial Maximum Volume (mL)**	**39.7 ± 15.5**	**34.9 ± 8.4**	**0.049**
Left Atrial Minimum Volume (mL)	13.8 ± 7.9	14.7 ± 5.7	0.548
Left Atrial Pre-Atrial Contraction Volume (mL)	23.7 ± 13.1	22.1 ± 7.6	0.461
**Reservoir Strain (%)**	**29.1 ± 9.7**	**34.2 ± 12.7**	**0.039**
Conduit Strain (%)	−17.6 ± 7.8	−19.4 ± 6.1	0.215
**Contractile Strain (%)**	**−11.6 ± 4.7**	**−14.7 ± 6.6**	**0.021**
Left Atrial Volume Index (mL/m^2^)	21.1 ± 7.8	18.7 ± 4.8	0.068
Left Atrial Contraction Index	0.16 ± 0.08	0.13 ± 0.05	0.291
**Left Atrial Ejection Fraction**	**0.66 ± 0.09**	**0.57 ± 0.13**	**<0.001**
**Left Atrial Expansion Index**	**2.22 ± 0.96**	**1.67 ± 1.01**	**0.005**
**Passive Emptying Fraction**	**0.42 ± 0.14**	**0.36 ± 0.13**	**0.043**
**Active Emptying Fraction**	**0.41 ± 0.13**	**0.32 ± 0.17**	**0.010**
**Left Atrial Stiffness Index**	**0.21 ± 0.12**	**0.18 ± 0.09**	**0.005**

Statistically significant parameters are highlighted in bold. Normal reference values for left atrial (LA) reservoir strain values were considered >35%. No universally accepted normal range for LA conduit and contractile strain. Reference ranges are provided for descriptive purposes only and were not used as diagnostic thresholds.

**Table 4:** Correlation analysis between clinical variables and LV and LA functional parameters.

Variable	LVGLS (r)	*p* Value	LA Reservoir Strain (r)	*p* Value	LA Conduit Strain (r)	*p* Value	LA EF (r)	*p* Value
FMF	−0.440	<0.001	0.221	0.039	−0.248	0.021	−0.353	<0.001
Diabetes mellitus	0.224	0.028	−0.295	0.006	—	—	—	—
Beta-blocker use	0.205	0.045	−0.228	0.034	—	—	—	—
Diuretic use	0.237	0.020	−0.197	0.068	—	—	—	—
Statin use	0.243	0.017	−0.227	0.034	—	—	—	—
Oral antidiabetic use	0.257	0.011	−0.310	0.003	—	—	—	—
Hyperlipidemia	—	—	−0.223	0.038	—	—	—	—
Hypertension	—	—	−0.193	0.089	—	—	—	—
Fasting glucose	—	—	−0.281	0.009	—	—	—	—
eGFR	—	—	—	—	—	—	0.274	0.005
HDL cholesterol	—	—	—	—	—	—	—	—
LDL cholesterol	—	—	—	—	—	—	—	—
Lymphocyte count	—	—	—	—	—	—	—	—
ACE inhibitor/ARB use	—	—	—	—	—	—	—	—
Calcium channel blocker use	—	—	—	—	—	—	—	—

Values are presented as correlation coefficients (r) and corresponding *p* values. Correlation analyses were performed using Pearson or Spearman correlation tests, as appropriate. LVGLS: left ventricular global longitudinal strain; LA: left atrial; EF: ejection fraction; FMF: Familial Mediterranean Fever; eGFR: estimated glomerular filtration rate; HDL: high-density lipoprotein; LDL: low-density lipoprotein; ARB: angiotensin receptor blocker. A dash (—) indicates non-significant associations (*p* ≥ 0.10).

**Table 5:** Correlation analysis between clinical variables and left atrial functional indices.

Variable	LA Expansion Index (r)	*p* Value	LA Passive EF (r)	*p* Value	LA Active EF (r)	*p* Value	LA Stiffness Index (r)	*p* Value
FMF	−0.274	0.005	−0.200	0.043	−0.254	0.010	—	—
Diabetes mellitus	—	—	—	—	0.313	0.001	0.369	<0.001
Beta-blocker use	—	—	—	—	—	—	—	—
Diuretic use	—	—	—	—	—	—	0.255	0.019
Statin use	—	—	—	—	0.252	0.010	—	—
Oral antidiabetic use	—	—	—	—	0.218	0.010	0.381	<0.001
Hyperlipidemia	—	—	—	—	—	—	—	—
Hypertension	—	—	—	—	—	—	0.292	0.007
Fasting glucose	—	—	—	—	0.245	0.013	0.384	<0.001
eGFR	0.226	0.023	—	—	—	—	—	—
HDL cholesterol	0.203	0.043	—	—	—	—	—	—
LDL cholesterol	0.210	0.035	—	—	—	—	—	—
Lymphocyte count	—	—	—	—	0.205	0.040	—	—
ACE inhibitor/ARB use	—	—	—	—	—	—	0.249	0.023
Calcium channel blocker use	—	—	—	—	—	—	0.390	<0.001

Values are presented as correlation coefficients (r) and corresponding *p* values. Correlation analyses were performed using Pearson or Spearman correlation tests, as appropriate. LA: left atrial; EF: emptying fraction; FMF: Familial Mediterranean Fever; eGFR: estimated glomerular filtration rate; HDL: high-density lipoprotein; LDL: low-density lipoprotein; ARB: angiotensin receptor blocker. A dash (—) indicates non-significant associations (*p* ≥ 0.10).

**Table 6:** Multivariable linear regression analyses of left ventricular and left atrial functional parameters.

Dependent Variable	Independent Variable	Unstandardized β	Standardized β	t Value	95% CI	*p* Value
LVGLS	FMF	−1.605	−0.374	−3.594	−2.566 to −0.958	<0.001
LA Reservoir Strain	Diabetes mellitus	−11.120	−0.297	−2.829	−18.938 to −3.302	<0.001
LA Conduit Strain	FMF	5.189	0.221	2.092	0.258 to 10.120	0.039
LA Ejection Fraction	FMF	−0.082	−0.327	−3.578	−0.127 to −0.036	<0.001
	eGFR	0.002	0.238	2.606	0.001 to 0.003	0.011
LA Expansion Index	FMF	−0.489	−0.236	−2.495	−0.879 to −0.100	0.014
	eGFR	0.012	0.212	2.246	0.001 to 0.023	0.027
	HDL cholesterol	0.016	0.191	2.029	0.001 to 0.031	0.045
LA Passive Emptying Fraction	FMF	−0.558	−0.274	−2.881	−0.943 to −0.174	0.005
LA Active Emptying Fraction	FMF	−0.577	−0.281	−2.926	−1.962 to −0.526	0.004

Regression models were constructed using the enter method. Only variables retained in the final models are shown. β indicates regression coefficient; CI, confidence interval; LVGLS, left ventricular global longitudinal strain; LA, left atrial; eGFR, estimated glomerular filtration rate; HDL, high-density lipoprotein; FMF, Familial Mediterranean Fever.

## Data Availability

The data presented in this study are available upon reasonable request from the corresponding author due to ethical and privacy restrictions.
